# Development of a novel human intestinal model to elucidate the effect of anaerobic commensals on *Escherichia coli* infection

**DOI:** 10.1242/dmm.049365

**Published:** 2022-04-28

**Authors:** Conor J. McGrath, Edgaras Laveckis, Andrew Bell, Emmanuelle Crost, Nathalie Juge, Stephanie Schüller

**Affiliations:** 1 Department of Clinical Medicine, Norwich Medical School, University of East Anglia, Norwich NR4 7UQ, UK; 2 Gut Microbes and Health Programme, Quadram Institute Bioscience, Gut Microbes and Health Institute Strategic Programme, Norwich NR4 7UQ, UK

**Keywords:** EPEC, *Limosilactobacillus reuteri*, *Ruminococcus gnavus*, Colonization resistance, Microbiota, Intestinal epithelium, Mucus, Model system

## Abstract

The gut microbiota plays a crucial role in protecting against enteric infection. However, the underlying mechanisms are largely unknown owing to a lack of suitable experimental models. Although most gut commensals are anaerobic, intestinal epithelial cells require oxygen for survival. In addition, most intestinal cell lines do not produce mucus, which provides a habitat for the microbiota. Here, we have developed a microaerobic, mucus-producing vertical diffusion chamber (VDC) model and determined the influence of *Limosilactobacillus reuteri* and *Ruminococcus gnavus* on enteropathogenic *Escherichia coli* (EPEC) infection. Optimization of the culture medium enabled bacterial growth in the presence of mucus-producing T84/LS174T cells. Whereas *L. reuteri* diminished EPEC growth and adhesion to T84/LS174T and mucus-deficient T84 epithelia, *R. gnavus* only demonstrated a protective effect in the presence of LS174T cells. Reduced EPEC adherence was not associated with altered type III secretion pore formation. In addition, co-culture with *L. reuteri* and *R. gnavus* dampened EPEC-induced interleukin 8 secretion. The microaerobic mucin-producing VDC system will facilitate investigations into the mechanisms underpinning colonization resistance and aid the development of microbiota-based anti-infection strategies.

This article has an associated First Person interview with the first author of the paper.

## Abstract (precis)

**Summary:** The new model system established in this study enables culture of human mucus-producing intestinal epithelia with oxygen-sensitive gut commensals and characterization of their effects on enteropathogenic *E. coli* infection.

## INTRODUCTION

Enteropathogenic *E. coli* (EPEC) is a major foodborne pathogen causing an estimated 120,000 deaths per year globally and affecting predominantly children under 5 years of age (Kirk et al., 2015; Lanata et al., 2013). The pathogenesis of EPEC is characterized by the formation of attaching/effacing (A/E) lesions, including tight adherence to the intestinal epithelium, microvillous effacement and actin polymerization beneath adherent bacteria (Knutton et al., 1989). A/E lesions are mediated by a type III secretion system (T3SS), which injects bacterial effector proteins into the host cell (Croxen and Finlay, 2010). Initial stages involve the establishment of a filamentous tube consisting of the EPEC T3S protein EspA, followed by translocation of EspB and EspD forming a pore in the host cell membrane (Garmendia et al., 2005). Translocated proteins include the translocated intimin receptor (Tir), which mediates intimate bacterial binding and an arsenal of additional effector proteins that interfere with host cell function, including epithelial permeability, water and ion transport and innate immune response, all of which contribute to the development of diarrhoea (Viswanathan et al., 2009).

Treatment of EPEC diarrhoea remains problematic owing to frequent failure to respond to oral rehydration therapy (Fagundes-Neto et al., 1996). In addition, multi-drug resistance is widespread among EPEC and use of antibiotics enhances the risk for development of persistent diarrhoea and malnutrition (Nguyen et al., 2005; Sarker et al., 2017; Eltai et al., 2020). Therefore, alternative treatment and prevention strategies are needed. In recent years, growing evidence has confirmed an important role of the gut microbiota in protecting against enteric infections and several mechanisms of colonization resistance have been unravelled (Sassone-Corsi and Raffatellu, 2015). These include direct interactions between commensal and pathogenic organisms (e.g. competition for nutrients, production of antimicrobials) and indirect effects of the microbiota on pathogen colonization via modulation of the host (e.g. strengthening of epithelial barrier function, stimulation of innate and adaptive immunity). Some of these mechanisms have been confirmed in EPEC infection of colon carcinoma cell lines, which demonstrated inhibition of EPEC adhesion and restoration of epithelial barrier function by lactobacilli and *E. coli* Nissle 1917 (Bernet et al., 1994; Sherman et al., 2005; Kleta et al., 2014; Zyrek et al., 2007) and in *in vitro* organ culture showing that *Limosilactobacillus reuteri* ATCC PTA 6475 and ATCC 53608 binding to the mucus layer resulted in decreased EPEC adherence to small intestinal biopsy epithelium (Walsham et al., 2016).

As most gut commensals are sensitive to oxygen, conventional cell culture models in air are limited with respect to determining the influence of members of the gut microbiota on EPEC infection of human intestinal epithelium. In addition, most commonly used intestinal epithelial cell lines (e.g. Caco-2, T84) are derived from absorptive enterocytes/colonocytes, representing the major cell type in the intestine. These cells do not produce mucus, which is secreted by specialized goblet cells and provides a habitat and nutrient source for the microbiota (Juge, 2012; Etienne-Mesmin et al., 2019; Navabi et al., 2013; Martens et al., 2018). In this study, we have adapted a vertical diffusion chamber (VDC) system, which allows infection of polarized intestinal epithelia under microaerobic conditions (Schüller and Phillips, 2010; McGrath and Schüller, 2021), for co-culture with the gut commensal strains *L. reuteri* ATCC PTA 6475 and *Ruminococcus gnavus* ATCC 35913. *L. reuteri* is a gut symbiont with mucin-binding capacity (MacKenzie et al., 2010), which has been shown to influence EPEC adhesion in *in vitro* organ culture (Walsham et al., 2016). *L. reuteri* has been studied as a model for host specialization across vertebrates (Frese et al., 2011) and earlier work reported that *L. reuteri* was the predominant autochthonous *Lactobacillus* species in infants as well as in adults (Reuter, 2001). *R. gnavus* is a prominent member of the human gut microbiota, where it belongs to the 57 species detected in more than 90% of human faecal samples (Qin et al., 2010). *R. gnavus* strains have been shown to adapt to the mucus layer by foraging on mucin glycan epitopes (Bell et al., 2019). Using the VDC model, we have investigated the effect of mucus-associated aerotolerant *L. reuteri* and anaerobic *R. gnavus* on EPEC growth, adherence, T3S and host inflammatory response.

## RESULTS

### Optimization of VDC culture medium

**Fig. 1.**
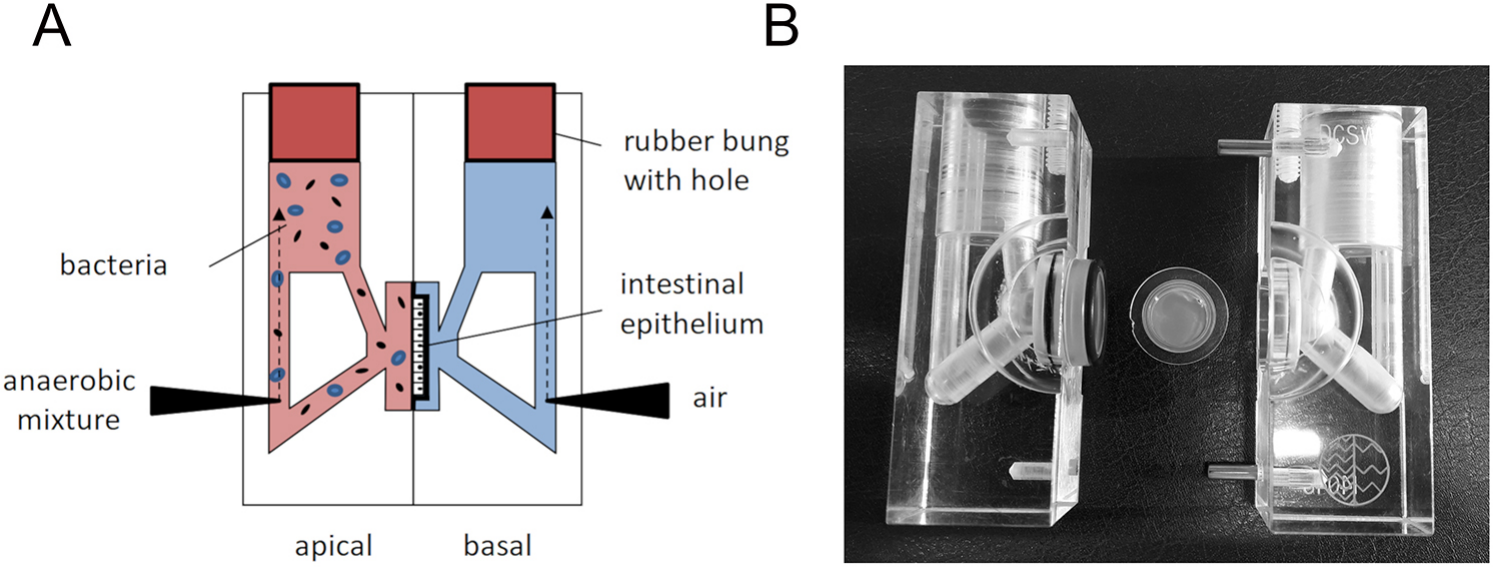
**Set-up of the VDC system.** (A) Schematic of the assembled VDC unit with cell monolayer in the middle. The apical medium (pink) is maintained anaerobically and inoculated with bacteria whereas the basal medium (blue) is gassed with air/CO_2_. Both half chambers are closed with a rubber bung with a central hole to maintain oxygen levels. (B) Photograph of VDC half chambers and Snapwell filter insert containing the cell monolayer (filter diameter=1.2 cm).

**Fig. 2.**
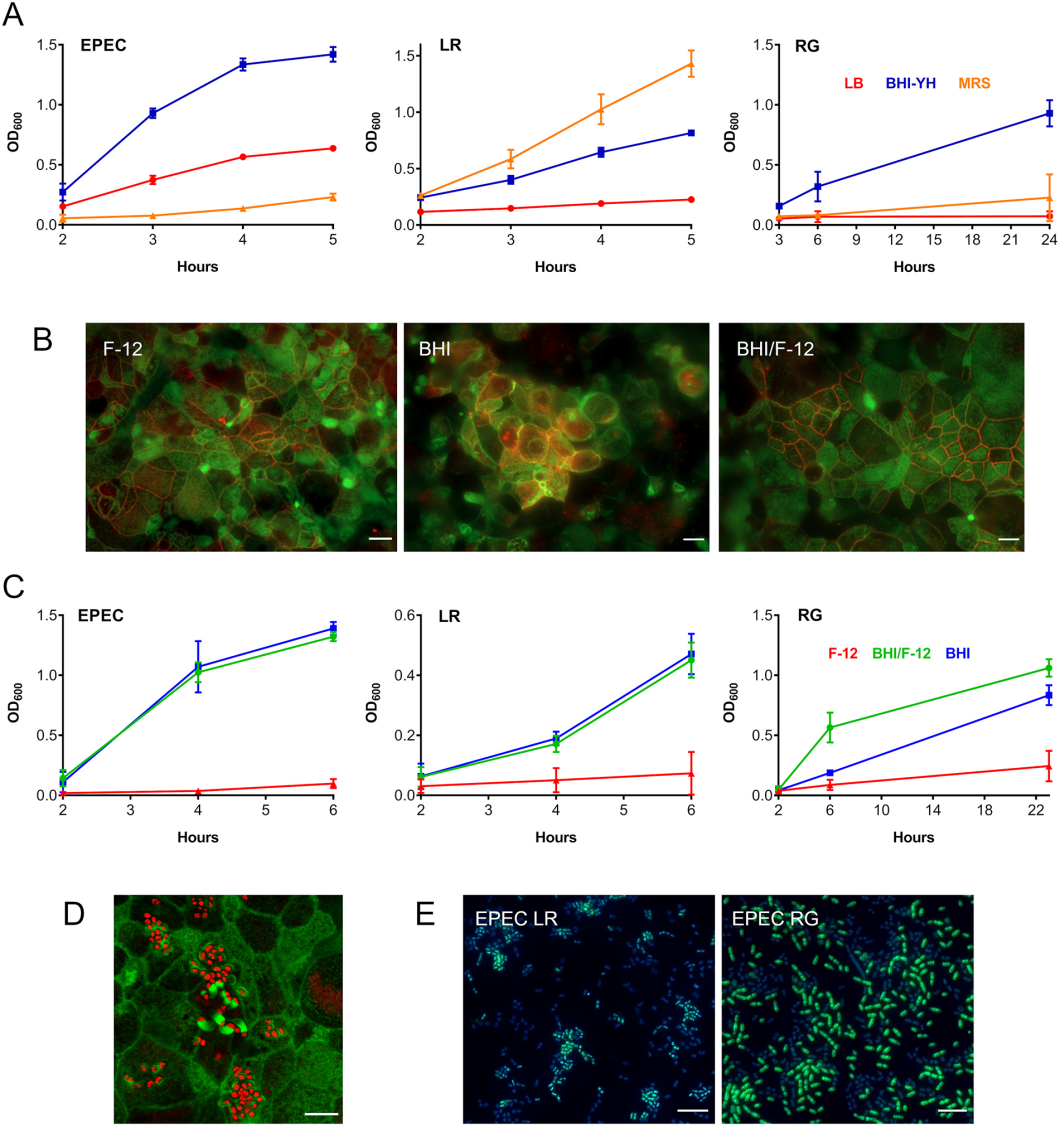
**Optimization of the apical VDC medium for co-incubation of EPEC, *L. reuteri* or *R. gnavus* and human T84 cell epithelia.** (A) Growth of EPEC, *L. reuteri* (LR) and *R. gnavus* (RG) in LB, MRS and BHI-YH broth. Bacteria were incubated in the VDC without host cells and OD_600_ was monitored over 5 h (EPEC and *L. reuteri*) or 24 h (*R. gnavus*). Mean±s.d., *n*=4. (B) Influence of BHI-YH medium on intestinal epithelial integrity and barrier function. Differentiated T84 cells were incubated in the VDC for 22 h with BHI-YH (BHI), DMEM/F-12 (F-12) or a 1:1 mixture of both media (BHI/F-12) on the apical side and DMEM/F-12 on the basal side. Epithelia were stained for F-actin (green) and occludin (red). Scale bars: 10 µm. Images are representative of *n*=6. (C) Growth of EPEC, *L. reuteri* and *R. gnavus* in BHI-YH (BHI), DMEM/F-12 (F-12) and BHI-YH/F-12 medium (BHI/F-12). Bacteria were incubated in the VDC without host cells and OD_600_ was monitored over 6 h (EPEC and *L. reuteri*) or 23 h (*R. gnavus*). Mean±s.d., *n*=4. (D) EPEC A/E lesion formation in T84 cells after 4 h incubation in apical BHI-YH/F-12 medium. Cells and EPEC were stained with F-actin (green) and DAPI (red), respectively. Scale bar: 5 µm. Images are representative of *n*=6. (E) Optimization of bacterial inocula for co-incubations. EPEC was cultured in the VDC with different ratios of *L. reuteri* or *R. gnavus* for 4 h. Culture composition was evaluated by immunofluorescence staining for *R. gnavus* NanH or *L. reuteri* CmbA (green) and counterstaining of all bacteria with DAPI (blue). Scale bars: 5 µm. Images are representative of *n*=6.

The bacteria used in our study have different nutrient requirements and thus are routinely grown in different culture media. Whereas EPEC is generally maintained in Lysogeny broth (LB), *R. gnavus* and *L. reuteri* are routinely cultured in Brain Heart Infusion broth supplemented with yeast extract and haemin (BHI-YH) and de Man, Rogosa and Sharpe (MRS) broth, respectively. To determine a suitable medium composition for the co-culture of EPEC prototype strain E2348/69 with *R. gnavus* ATCC 35913 or *L. reuteri* ATCC PTA 6475, growth of all three strains was quantified in LB, MRS and BHI-YH medium. This was performed in the microaerobic VDC system employed throughout this study (Fig. 1), but without the presence of host epithelia. As shown in Fig. 2A, the VDC supported growth of all strains, including the strict anaerobe *R. gnavus*. The highest absorbance for EPEC and *R. gnavus* was obtained in BHI-YH medium, whereas *L. reuteri* grew best in MRS broth. BHI-YH medium was chosen for subsequent experiments as it supported growth of all three organisms.

To investigate the effect of BHI-YH on the integrity and barrier function of human intestinal epithelia, polarized T84 monolayers were incubated with BHI-YH, DMEM/F-12 (culture medium for T84 cells) and a 1:1 mixture of both media (BHI-YH/F-12) on the apical side of the VDC, whereas DMEM/F-12 was used in the basal compartment. Epithelial structure and barrier function were assessed by immunofluorescence staining for filamentous actin and the tight junction protein occludin. In addition, the transepithelial electrical resistance (TEER) was determined before and after incubation. Although epithelial cells were well preserved in DMEM/F-12 and BHI-YH/F-12 medium, cell rounding, loss of apical brush border and disruption of tight junctions was observed in T84 monolayers cultured in BHI-YH medium for 4 and 22 h (Fig. 2B, images shown for 22 h). Compromised epithelial barrier function in BHI-YH medium was also confirmed by a significant reduction in TEER (Fig. S1).

Next, growth of EPEC, *L. reuteri* and *R. gnavus* was examined in BHI-YH, DMEM/F-12 and BHI-YH/F-12 in the VDC in the absence of host epithelia. Although none of the strains showed considerable growth in DMEM/F-12, similar OD_600_ values were observed between cultures maintained in BHI-YH and BHI-YH/F-12 (Fig. 2C). In addition, EPEC A/E lesion formation was evident after 4 h of incubation in BHI-YH/F-12 medium (Fig. 2D). Therefore, BHI-YH/F-12 was selected as apical medium for subsequent VDC experiments.

### Adjustment of bacterial inocula

As *L. reuteri* ATCC PTA 6475 and *R. gnavus* ATCC 35913 strains exhibited slower growth in BHI-YH/F-12 than EPEC E2348/69, bacterial inocula required adjustment to avoid EPEC overgrowth in bacterial co-incubation experiments. To this end, EPEC was cultured in the VDC without host cells with different ratios of *L. reuteri* or *R. gnavus*. Co-cultures were evaluated by immunofluorescence staining using antibodies specific for *R. gnavus* ATCC 35913, *L. reuteri* ATCC PTA 6475 and EPEC. The results showed that inocula of approximately 6.25×10^4^ colony-forming units (CFU)/ml of EPEC, 7×10^7^ CFU/ml of *L. reuteri* and 9.8×10^6^ CFU/ml of *R. gnavus* resulted in similar numbers of EPEC and commensal strains after 4 h of incubation (Fig. 2E). Therefore, these inocula were employed in the rest of the study.

### Introduction of mucus-producing cells

**Fig. 3.**
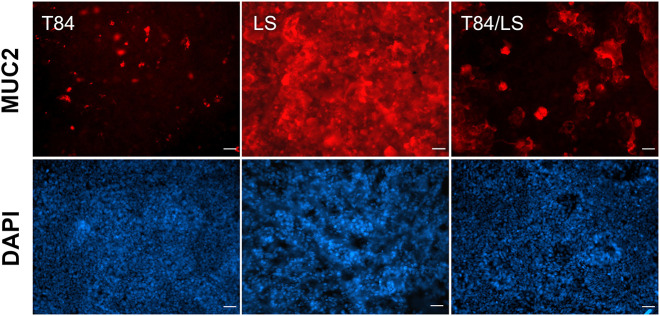
**Mucus production by T84, LS174T and mixed epithelia.** Cell monolayers were incubated in the VDC for 4 h and stained with rabbit anti-MUC2 (red). Cell nuclei were counterstained with DAPI (blue). Scale bars: 25 µm. Images are representative of *n*=4.

In order to introduce a mucus layer, T84 cells were co-cultured with mucus-producing LS174T cells at a ratio of 10:1. Whereas T84 cells demonstrated low levels of MUC2 staining localized to few individual cells, LS174T epithelia exhibited a continuous mucus layer (Fig. 3). An intermediate phenotype was observed in T84/LS174T cultures, which produced patches of secreted mucus (Fig. 3 and Fig. S2). In addition, T84 cells formed tight monolayers with high epithelial barrier function (TEER >1000 Ω × cm^2^), which was considerably reduced in LS174T and T84/LS174T epithelia (15 and 30-60 Ω × cm^2^, respectively).

**Fig. 4.**
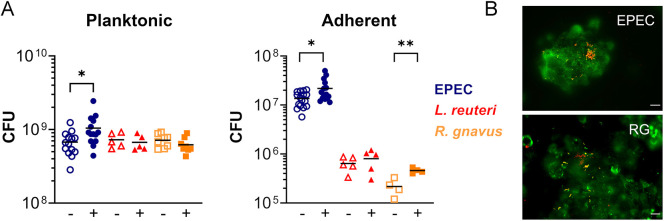
**Influence of mucin-producing LS174T cells on growth and adherence of EPEC, *L. reuteri* and *R. gnavus*.** Bacterial strains were incubated in the VDC with T84 (−) or T84/LS174T (+) epithelia for 4 h. (A) Growth of planktonic bacteria and epithelial adherence were evaluated by quantifying CFU in apical media and cell lysates, respectively. Shown are individual data points. Means are indicated by a line. Data were analysed using unpaired *t*-test, *P<0.05, **P<0.01. (B) Binding of EPEC and *R. gnavus* (RG) to T84/LS174T epithelia. Bacteria are labelled in red and LS174T cells are stained with anti-MUC2 (green). Scale bars: 10 µm.

Next, we determined how the inclusion of mucin-producing LS174T cells affected planktonic growth and adherence of all bacterial strains. As shown in Fig. 4, similar levels of planktonic growth were observed for all three strains, indicating successful adjustment of inocula. Although EPEC proliferation was significantly enhanced in the presence of T84/LS174T monolayers, no effect was observed for *L. reuteri* and *R. gnavus*. Interestingly, the addition of LS174T cells resulted in significantly increased numbers of adherent EPEC and *R. gnavus* but not *L. reuteri*, and immunostaining revealed preferential binding to LS174T cells (Fig. 4).

**Fig. 5.**
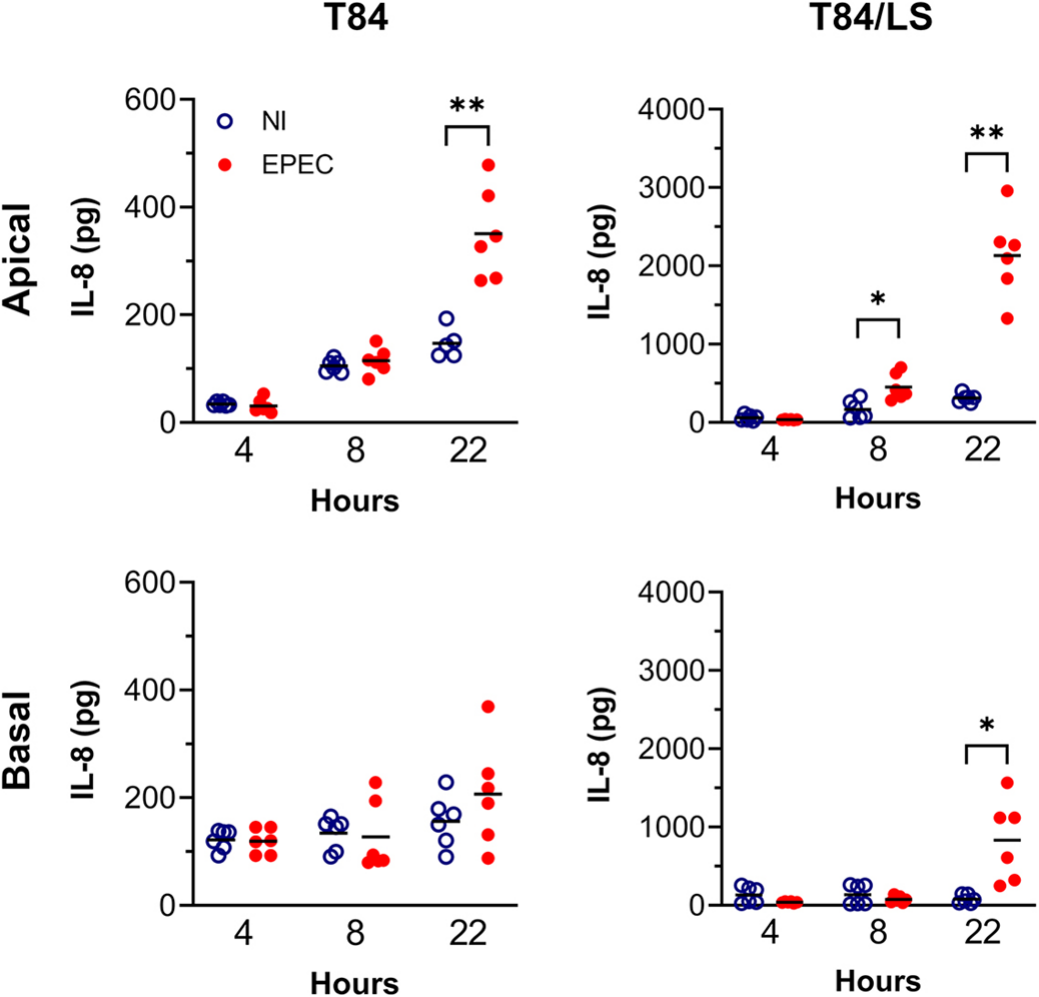
**IL-8 secretion in response to EPEC infection by T84 and T84/LS174T epithelia.** Cell monolayers were incubated with EPEC or left non-infected (NI) for 4, 8 or 22 h in the VDC. Medium containing gentamicin (50 µg/ml) was added after 4 h to prevent bacterial overgrowth. IL-8 in apical and basal supernatants was quantified by ELISA and expressed as total pg per chamber. Shown are individual data points with means indicated by a line. Data were analysed by two-way ANOVA with Sidak's multiple comparison test, *P<0.05, **P<0.01.

In addition, we investigated the effect of LS174T cells on EPEC-induced secretion of the pro-inflammatory chemokine interleukin8 (IL-8). To prevent EPEC overgrowth and loss of epithelial barrier function (McNamara et al., 2001), bacteria were killed by addition of gentamicin after 4 h of incubation. As shown in Fig. 5, EPEC-stimulated IL-8 secretion was considerably higher in T84/LS174T compared with T84 epithelia with a mean value of 2264 versus 346 pg IL-8 in apical supernatants after 22 h of infection. Both T84 and mixed cultures exhibited similar kinetics with maximal IL-8 secretion after 22 h. In addition, more IL-8 was released into apical than basal compartments. Epithelial barrier function was maintained throughout the 22 h incubation period as evidenced by TEER values of approximately 800 Ω × cm^2^ in EPEC-infected and control T84 epithelia (Fig. S3). As noted before, T84/LS174T monolayers exhibited very low TEER (∼45 Ω × cm^2^).

### Effect of *L. reuteri* and *R. gnavus* on EPEC pathogenesis

#### EPEC growth and adherence

**Fig. 6.**
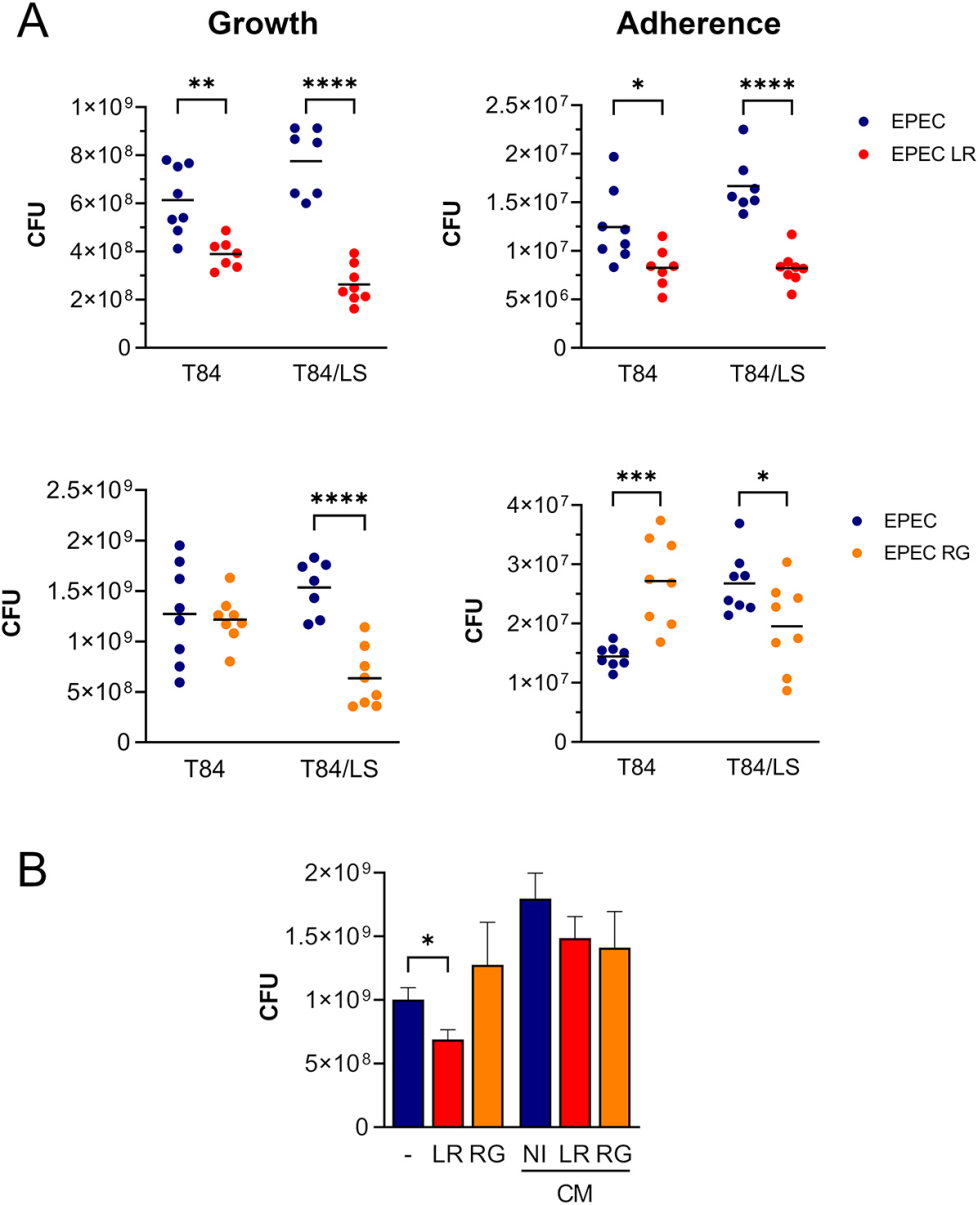
**Influence of *L. reuteri* and *R. gnavus* on EPEC planktonic growth and epithelial adhesion.** (A) T84 and T84/LS174T epithelia were incubated with EPEC and *L. reuteri* (LR) or *R. gnavus* (RG) using previously established inocula. Cells were cultured with EPEC alone as control. After 4 h of incubation in the VDC, EPEC growth and binding to host epithelia were quantified by determining CFU. Shown are total CFU in apical media and cell lysates, respectively. Data are plotted as individual data points with means indicated by a line. Statistical analysis was performed by two-way ANOVA with Sidak's multiple comparison test, *P<0.05, **P<0.01, ***P<0.001, ****P<0.0001. (B) EPEC was incubated with *L. reuteri* (LR), *R. gnavus* (RG) or medium alone (−) in the VDC without host cells for 4 h. In addition, EPEC cultures were performed with conditioned media (CM) from LR, RG or medium control (NI). EPEC growth was quantified by CFU. Data are shown as mean±s.e.m. of *n*=4. Statistical analysis was performed by one-way ANOVA with Tukey's multiple comparison test, *P<0.05.

Having established the mucin-producing T84/LS174T VDC model, we examined the influence of *L. reuteri* ATCC PTA 6475 and *R. gnavus* ATCC 35913 strains on EPEC growth and host cell adherence. As observed previously, planktonic growth and adherence of EPEC was generally higher in T84/LS174T compared with T84 cultures (Fig. 6A). Addition of *L. reuteri* resulted in significantly reduced numbers of planktonic and adherent EPEC in both T84 and T84/LS174T monolayers, although the difference was more pronounced in T84/LS174T epithelia. Similarly, the presence of *R. gnavus* significantly inhibited EPEC growth and binding to T84/LS174T cells (Fig. 6A). Pearson correlation analysis revealed no significant association between reduced proliferation and adherence in the presence of *L. reuteri* (r=−0.076, *P*=0.8584 for T84; r=−0.001, *P*=0.9974 for T84/LS174T cultures), but a significant positive correlation for co-cultures with *R. gnavus* (r=0.807, *P*=0.015). In contrast, no difference in luminal EPEC growth in the presence or absence of *R. gnavus* was observed in T84 cultures, and EPEC adhesion was even significantly enhanced in cultures with *R. gnavus*. Epithelial integrity of neither T84 nor T84/LS174T epithelia was affected by co-culture with EPEC and commensals (Fig. S4). Notably, incubation of EPEC with conditioned media from *L. reuteri* or *R. gnavus* cultures did not influence EPEC growth (Fig. 6B).

#### EPEC type III secretion

**Fig. 7.**
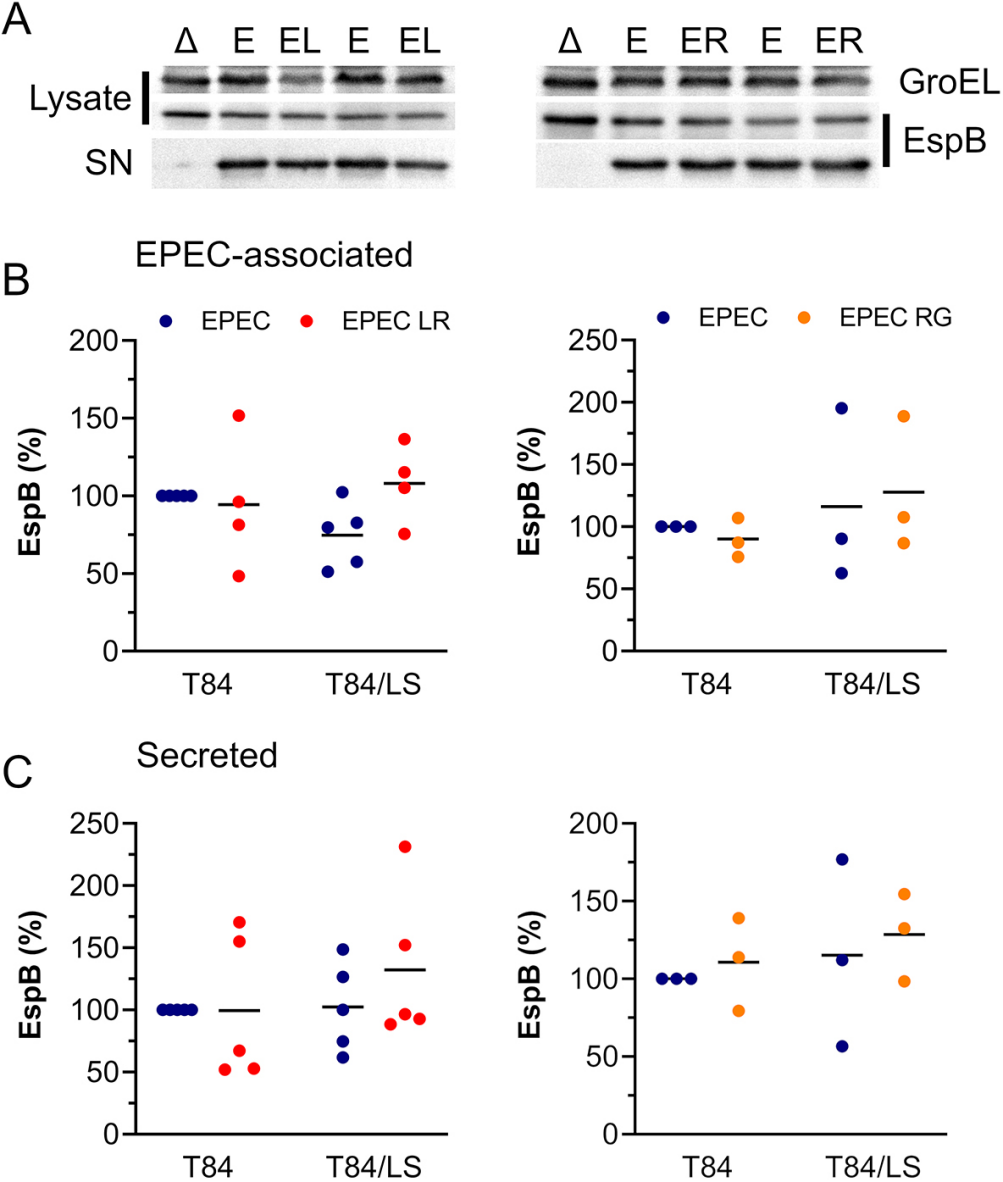
**EPEC EspB production and secretion in the presence of *L. reuteri* and *R. gnavus*.** (A-C) T84 and T84/LS174T epithelia were incubated with EPEC alone (E) or in co-culture with *L. reuteri* (EL) or *R. gnavus* (ER) for 4 h. T84/LS174T cells were also inoculated with EPEC Δ*escN* (Δ) to control for T3S. Apical media were sampled, and bacteria separated from supernatants by centrifugation. Bacterial lysates and concentrated supernatants (SN) were separated by 12% SDS-PAGE, and EspB was detected by western blotting. Membranes were re-probed for GroEL as housekeeping control (A). Bands were quantified by densitometry, and EspB signals were normalized to GroEL controls. EspB levels in EPEC-associated (B) and supernatant (C) fractions are expressed as percentage relative to EPEC-infected T84 cells. Data are plotted as individual data points with means indicated by a line. Statistical analysis was performed by two-way ANOVA with Tukey's multiple comparison test.

We next evaluated whether reduced EPEC adherence in the presence of *L. reuteri* or *R. gnavus* was associated with impaired expression or secretion of the pore-forming protein EspB. VDC experiments with T84 and T84/LS174T epithelia were performed with EPEC alone and in co-culture with *L. reuteri* or *R. gnavus*. In addition, the T3S-deficient EPEC mutant Δ*escN* was included in the T84/LS174T cell-based VDC to control for efficient separation of EPEC-associated and secreted EspB. Levels of EspB in bacterial cells and supernatants were quantified by western blotting. Membranes were also probed for the *E. coli* housekeeping protein GroEL to allow for adjustment of EPEC numbers. No anti-EspB or GroEL cross-reaction was observed between lysates from *L. reuteri* and *R. gnavus* (data not shown). Although EspB was only apparent in EPEC Δ*escN* bacterial cells, EspB-specific bands were also evident in supernatant fractions of wild-type EPEC, confirming EspB secretion into the medium (Fig. 7A). No significant differences in levels of bacterial and secreted EspB were detected in the presence of *L. reuteri* or *R. gnavus*, as shown by densitometric analyses and normalization to GroEL (Fig. 7B,C).

#### Influence of *L. reuteri* and *R. gnavus* on EPEC-induced inflammation

**Fig. 8.**
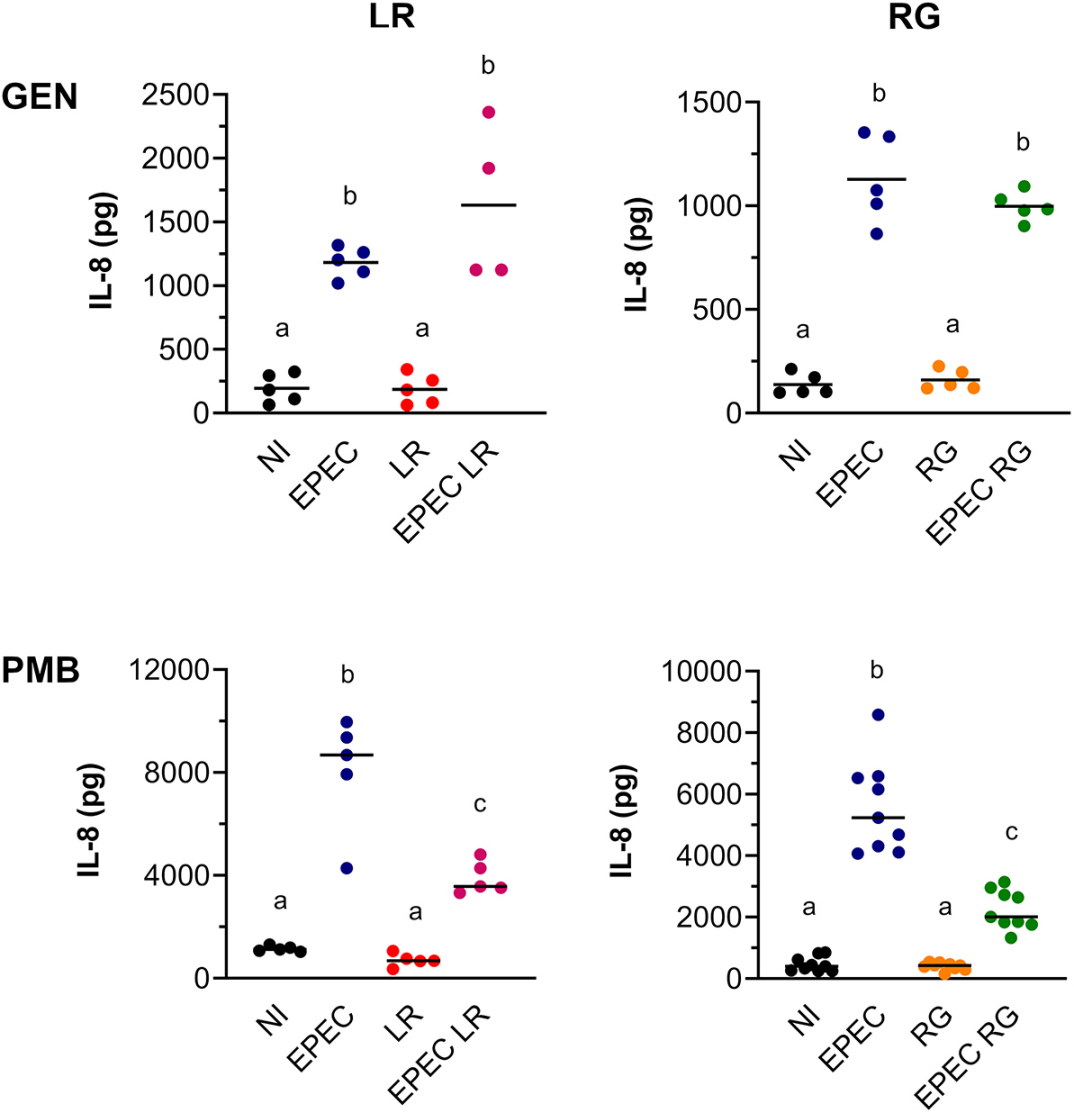
**Effect of *L. reuteri* and *R. gnavus* on IL-8 release during EPEC infection.** T84/LS174T epithelia were incubated with EPEC, *L. reuteri* (LR), EPEC and *L. reuteri* (EPEC LR) or left non-infected (NI) (left). In addition, incubations were performed with EPEC, *R. gnavus* (RG), EPEC and *R. gnavus* (EPEC RG) and NI controls (right). After 4 h, media in apical chambers were exchanged for media containing 50 µg/ml gentamicin (GEN, top) or 16 µg/ml polymyxin B (PMB, bottom), and cultures were continued for 18 h. IL-8 levels in apical supernatants were determined by ELISA. Data are shown as individual data points with means indicated by a line. Statistical analysis was performed by one-way ANOVA with Tukey's multiple comparison test. Groups with different letters are significantly different.

To investigate the effect of *L. reuteri* ATCC PTA 6475 and *R. gnavus* ATCC 35913 strains on EPEC-induced IL-8 secretion, T84/LS174T monolayers were incubated with EPEC in the presence or absence of *L. reuteri* or *R. gnavus*. In addition, epithelia were cultured with each commensal strain alone or left non-infected (NI) to assess IL-8 baseline secretion. To prevent bacterial overgrowth, apical media were replaced with F-12/BHI-YH containing gentamicin and incubations were continued for a further 18 h. IL-8 release into the supernatant was determined by enzyme-linked immunosorbent assay (ELISA). Although EPEC infection alone resulted in significantly enhanced IL-8 levels compared with NI controls (***P<0.001), this induction was not affected by co-culture with *L. reuteri* or *R. gnavus* (Fig. 8, top). Notably, neither *L. reuteri* nor *R. gnavus* alone stimulated IL-8 release from T84/LS174T epithelia. Given that *L. reuteri* and *R. gnavus* grow more slowly in the VDC system compared with EPEC (Fig. 2C), it is possible that more than 4 h are required for the establishment of immunomodulatory effects. Therefore, the use of polymyxin B, which specifically targets Gram-negative bacteria, was considered. Whereas gentamicin (50 µg/ml) prevented growth of all bacterial strains, polymyxin B inhibited replication of Gram-negative EPEC at a concentration of 4 µg/ml but did not affect Gram-positive *L. reuteri* and *R. gnavus* at concentrations of up to 16 µg/ml (Fig. S5). The addition of polymyxin B at 4 h post-infection selectively inhibited EPEC growth while maintaining multiplication of *L. reuteri* and *R. gnavus*. Under these conditions, both *L. reuteri* and *R. gnavus* significantly reduced the IL-8 response of T84/LS174T epithelia to EPEC infection (Fig. 8, bottom; ***P<0.001 and ****P<0.0001, respectively). Similar to previous experiments with gentamicin, culture with commensal strains alone did not stimulate an IL-8 response above baseline levels.

## DISCUSSION

The important role of the gut microbiota in protecting against enteric infections has prompted a rise in research into the underlying mechanisms of colonization resistance and their potential applications for new therapeutic approaches (Sassone-Corsi and Raffatellu, 2015; Ducarmon et al., 2019). The development of suitable experimental model systems to decipher pathogen-microbiota-host interactions *in vitro* poses a challenge, largely because of difficulties in simulating the microaerobic environment at the gut mucosa and supporting the survival of the mostly oxygen-sensitive gut microbiota (Albenberg et al., 2014; Thursby and Juge, 2017). Therefore, *in vivo* studies in small animal models are often employed, and colonization resistance against EPEC infection has been explored in mice infected with the natural murine A/E pathogen *Citrobacter rodentium* (Mullineaux-Sanders et al., 2018). This work showed that *Lactobacillus* species and *Bifidobacterium breve* reduce *C. rodentium* colonization, epithelial barrier dysfunction and host inflammation (Rodrigues et al., 2012; Fanning et al., 2012). Although *C. rodentium* shares many features with EPEC, their tissue tropism, histopathology and clinical symptoms after infection differ (Dupont et al., 2016).

In addition, there are intrinsic differences in the microbiota of mice and humans, and, as a result of co-evolution, host-microbe crosstalk is highly host specific (Frese et al., 2011; Nguyen et al., 2015). This has driven the recent development of microaerobic human intestinal epithelial models, including the Human oxygen-Bacteria anaerobic (HoxBan) (Sadaghian Sadabad et al., 2015), host–microbiota interaction (HMI) (Marzorati et al., 2014), the Human Microbial Crosstalk (HuMiX) (Shah et al., 2016) and anaerobic Gut-on-a-Chip (Shin et al., 2019; Jalili-Firoozinezhad et al., 2019) systems. In the HoxBan model, the bacteria are contained within a solid agar column, thereby restricting dynamic interactions between pathogens, commensals and the host epithelium. In the HMI and HuMiX systems, bacteria are separated from the host epithelium by a porous membrane, which prevents EPEC adhesion and A/E lesion formation. Although the microfluidic Gut-on-a-Chip model fulfils all criteria for co-culturing differentiated human intestinal epithelia with EPEC and oxygen-sensitive commensal bacteria, the operation of this system is associated with considerable costs and technical sophistication requiring extensive training. Therefore, we have adapted an Ussing chamber-based VDC model, which has been successfully employed to investigate pathogenesis of anaerobic intestinal pathogens, including *Helicobacter pylori* (Cottet et al., 2002), *Campylobacter jejuni* (Mills et al., 2012), and *Clostridioides difficile* (Jafari et al., 2016; Anonye et al., 2019). In addition, the VDC system has been used to determine the influence of the low-oxygen environment in the gut on virulence of enterohaemorrhagic and enteroaggregative *E. coli* (Schüller and Phillips, 2010; Tran et al., 2014; Ellis et al., 2019). In this study, we demonstrate that oxygen levels in the VDC system are sufficiently low to support growth of the strictly anaerobic gut symbiont *R. gnavus*. This is in agreement with previous work that reported multiplication of the anaerobic microbiota species *Bacteroides dorei* in the VDC system (Anonye et al., 2019). Oxygen levels in the apical VDC compartment have been determined as 1-1.7% of atmospheric pressure (Schüller and Phillips, 2010), which is similar to the oxygen pressure at the gut mucosal surface (He et al., 1999).

In addition to microaerobic conditions, the VDC presented here includes mucin-producing cells. Similar to goblet cells in the human intestinal epithelium, LS174T cells produce the major secreted mucin glycoprotein MUC2 (van Klinken et al., 1996; Johansson et al., 2011). In the human small intestine, goblet cells represent 8-10% of epithelial cells, which is similar to the ratio applied in this study (Umar, 2010). Inclusion of LS174T cells in the T84 monolayer resulted in lower epithelial barrier function, which is more similar to the resistance of native human colonic epithelium (approximately 95 Ω × cm^2^) (Schmitz et al., 2000). Notably, EPEC growth was enhanced in the presence of T84/LS174T epithelia; this may be mediated by the mucinase SslE, which has been shown to promote growth of extra-intestinal *E. coli* in mucus derived from human intestinal HT29-MTX cells (Valeri et al., 2015). In addition to EPEC growth, host cell adhesion was increased in the presence of LS174T cells; this may be mediated by H6 flagella, which have been shown to bind bovine colonic mucus (Erdem et al., 2007).

*R. gnavus* exhibited similar planktonic growth but increased adhesion to T84/LS174T versus T84 cell monolayers. Given the ability of *R. gnavus* ATCC 35913 to utilize sialylated mucins as a carbon source by means of the intramolecular *trans*-sialidase NanH (Crost et al., 2016), the lack of enhanced growth in the presence of MUC2-producing LS174T cells is surprising. However, the apical medium used in our study (F12/BHI-YH) might provide more easily accessible carbon sources (e.g. glucose), so that a growth advantage might not be evident after 4 h of incubation. Alternatively, mucus degradation might be restricted to bacteria in the direct vicinity of the mucus layer, which might explain increased numbers of cell-associated *R. gnavus* in T84/LS174T epithelia versus T84 monocultures. Interestingly, NanH also contains a carbohydrate-binding module and enables *R. gnavus* binding to intestinal mucus via sialic acid (Owen et al., 2017).

Clinical studies have suggested probiotic effects of *L. reuteri* in intestinal infection. In particular, administration of strains SD 2112 and DSM 17938 reduced the duration of infectious diarrhoea in children (Szajewska et al., 2014; Shornikova et al., 1997), and prophylactic treatment with *L. reuteri* DSM 17938 resulted in lower EPEC colonization in hospitalized infants (Savino et al., 2015). To elucidate the mechanisms underpinning colonization resistance of *L. reuteri* and other *Lactobacillus* strains against EPEC infection, aerobic cell and intestinal biopsy cultures have been performed, and competition for bacterial binding sites at the mucus layer and intestinal epithelium have been identified (Bernet et al., 1994; Sherman et al., 2005; Walsham et al., 2016). However, studies performed in the HuMiX model demonstrated considerable differences in the intestinal epithelial response to *Lactobacillus rhamnosus* GG (LGG) under aerobic versus anaerobic conditions (Shah et al., 2016). Notably, the latter exhibited high levels of concordance with clinical transcriptomic data from human intestinal biopsy samples after administration of LGG, thus demonstrating the importance of low oxygen levels for the reproduction of physiologically relevant host responses in the gut.

In the VDC system, co-culture with *L. reuteri* resulted in reduced growth of planktonic EPEC when grown with T84 or mixed epithelia. As conditioned media from *L. reuteri* failed to mimic this effect, competition for nutrients or production of toxic metabolites are unlikely mechanisms. In addition to the effect on growth, EPEC adhesion to T84 and T84/LS174T epithelia was diminished in the presence of *L. reuteri*. This is unlikely a direct consequence of lower numbers of planktonic EPEC, as indicated by correlation analysis. Interestingly, two recent studies discovered that commensal *Enterococcus faecalis* and *Bacteroides thetaiotaomicron* release proteases that degrade the translocon protein EspB of the EPEC-related A/E pathogen enterohaemorrhagic *E. coli* (EHEC) and thereby modulate T3S and A/E lesion formation (Cameron et al., 2019, 2018). However, no effect of *L. reuteri* on EPEC EspB expression and secretion was observed in our study, and therefore other potential mechanisms need to be explored.

A different effect was observed when EPEC was cultured with *R. gnavus* in the VDC system. Notably, reduced EPEC growth was only detected in the presence of T84/LS174T but not T84 epithelia or in the absence of host cells, suggesting that EPEC and *R. gnavus* may be competing for common nutrient sources derived from LS174T cells. Correlation analysis indicates that inhibition of EPEC replication by *R. gnavus* may result in lower adhesion to T84/LS174T epithelia. In addition, cell-associated *R. gnavus* could block EPEC-binding sites, which is supported by higher *R. gnavus* adhesion to T84/LS174T compared with T84 cells. Surprisingly, EPEC adherence to T84 epithelia was enhanced in the presence of *R. gnavus*. Previous examples of commensal bacteria promoting virulence of diarrhoeagenic *E. coli* include *B. thetaiotaomicron* and *E. faecalis*, which enhance EHEC T3SS gene expression and subsequent A/E lesion formation by producing succinate (Curtis et al., 2014). Although *R. gnavus* did not affect EPEC EspB secretion, we cannot exclude the possibility of the production of metabolites modulating expression of other T3SS components or adhesins.

EPEC infection is characterized by moderate inflammation and neutrophil influx in infected tissues, which is mediated by the pro-inflammatory chemokine IL-8 (Ulshen and Rollo, 1980; Baggiolini and Clark-Lewis, 1992). Accordingly, previous studies have demonstrated that EPEC infection stimulates IL-8 release from T84 cells and that this response is largely dependent on flagellin (Zhou et al., 2003; Ruchaud-Sparagano et al., 2007). In contrast, LS174T cells have been used less frequently in bacterial infection studies, but have been shown to secrete IL-8 after exposure to flagellin from *Salmonella* Typhimurium (Croix et al., 2011). Our data indicate an augmented IL-8 response in T84/LS174T versus T84 epithelia, which might be explained by higher numbers of planktonic and adherent EPEC and shedding of flagellin in the presence of LS174T cells. Interestingly, higher levels of IL-8 were detected in apical versus basal supernatants of EPEC-infected T84 and T84/LS174T monolayers. This is contradictory to findings in polarized Caco-2 cells, where apical EPEC infection resulted in predominantly basal IL-8 release (Ruchaud-Sparagano et al., 2007). However, previous work in our laboratory has shown vectorial IL-8 secretion in EHEC-infected T84 cells with apical EHEC exposure inducing apical IL-8 release whereas basolateral infection resulted in IL-8 secretion into the basal compartment (Lewis et al., 2016). Notably, co-culture with *L. reuteri* and *R. gnavus* reduced IL-8 secretion by EPEC-infected T84/LS174T epithelia in long-term incubations. This agrees with previous studies that have shown anti-inflammatory properties for *L. reuteri* in lipopolysaccharide (LPS)- or rotavirus-induced intestinal inflammation (Liu et al., 2010; Preidis et al., 2012). Recent work has identified a cell wall-associated glucorhamnan polysaccharide in the *R. gnavus* strain ATCC 29149 that stimulates TNF-α release by dendritic cells via activation of TLR4 (Henke et al., 2019). However, intestinal epithelial cells express very little TLR4 (Abreu et al., 2001), which might explain the lack of IL-8 secretion in T84/LS174T epithelia cultured with *R. gnavus* in our study. Co-culture with *R. gnavus* reduced IL-8 levels during EPEC infection, which may reflect our observations of decreased EPEC replication and adhesion.

In summary, we have established and applied a microaerobic, mucin-producing human intestinal culture system that supports the growth of oxygen-sensitive commensal bacteria and allows direct microbe-host contact. The VDC model will enable mechanistical analysis of colonization resistance against EPEC infection and thus aid in the development of gut microbiota-based therapies. In addition, the VDC can be adapted to explore colonization resistance in other enteric infections provided that a suitable co-culture medium for host cells and microbes can be identified.

## MATERIALS AND METHODS

### Bacterial strains and culture conditions

Bacterial strains used in this study were *L. reuteri* ATCC PTA 6475, *R. gnavus* ATCC 35913, EPEC O127:H6 E2348/69 and an isogenic *escN* deletion mutant (kindly provided by Gad Frankel, Imperial College London, UK). EPEC and *L. reuteri* were grown standing in LB (Formedium) and MRS broth (Oxoid), respectively, overnight at 37°C. *R. gnavus* was grown standing in an anaerobic cabinet (5% CO_2_, 10% H_2_ and 85% N_2_) overnight at 37°C in BHI-YH (Oxoid/Becton Dickinson). Kanamycin (50 µg/ml) was used for selection of EPEC Δ*escN*.

### Cell culture

All cell lines were freshly obtained from the European Collection of Authenticated Cell Cultures (ECACC) and regularly tested for mycoplasma contamination using the LookOut Mycoplasma qPCR Detection Kit (Sigma-Aldrich). T84 human colon carcinoma cells (ECACC 88021101) were cultured in Dulbecco's Modified Eagle's Medium/Nutrient F-12 Ham 1:1 (DMEM/F12) medium (Sigma-Aldrich) supplemented with 10% foetal bovine serum (Sigma-Aldrich) and 2.5 mM L-glutamine (Sigma-Aldrich) and used between passages 46-62. Mucin-producing LS174T human colon carcinoma cells (ECACC 87060401) were cultured in Dulbecco's Modified Eagle's Medium (DMEM) supplemented with 10% foetal bovine serum, 4 mM L-glutamine and 1× non-essential amino acids (Sigma-Aldrich). For VDC experiments, 5×10^5^ T84 cells were seeded out on polyester Snapwell filter inserts (12 mm diameter, 0.4 μm pore, Corning) coated with 10 µg/cm^2^ rat tail collagen type I (Sigma-Aldrich). For T84/LS174T co-cultures, 5.6×10^4^ LS174T cells were added (ratio 10:1). TEER was measured using an EndOhm chamber and EVOM resistance meter (World Precision Instruments) and values of >1000 Ω × cm^2^ after 10-13 days of cell differentiation indicated establishment of T84 epithelial barrier function. Cells were grown at 37°C in a 5% CO_2_ atmosphere.

### VDC incubations

A NaviCyte vertical diffusion chamber system (Harvard Apparatus) with Snapwell chambers was used in this study. For initial optimization experiments without host epithelia, VDC half chambers were assembled with empty Snapwell holders (filters were removed with a razor blade). Each VDC unit was filled with 8 ml of pre-reduced medium as indicated and gassed with an anaerobic mixture (90% N_2_, 5% H_2_ and 5% CO_2_) on both slides. For incubation of intestinal epithelia, Snapwell inserts with T84 or T84/LS174T monolayers were mounted between half chambers (for a detailed description, see McGrath and Schüller, 2021). Apical compartments were filled with 4 ml of pre-reduced BHI-YH, DMEM/F-12 or a mixture of both and maintained under anaerobic conditions. Basal chambers contained 4 ml of DMEM/F-12 and were perfused with 95% air, 5% CO_2_. Apical compartments were inoculated with bacteria as specified in the Results section and incubated for indicated time periods. For incubations of 8 or 22 h, apical media containing bacteria were removed after 4 h, and fresh media containing gentamicin (50 µg/ml) or polymyxin B (16 µg/ml) were added to prevent EPEC overgrowth. For preparation of conditioned media, *L. reuteri*, *R. gnavus* or BHI-YH/F-12 medium alone were incubated in the VDC without host cells for 4 h. Media were sampled and bacteria were removed by centrifugation (5000 ***g*** for 10 min) and filter sterilization. Conditioned media were used at a dilution of 1:2 in BHI-YH/F-12 in VDC experiments.

### Quantification of bacterial growth and adherence

Growth-curve analysis was performed in 96-well plates in media containing gentamicin and polymyxin B (Sigma-Aldrich) at the indicated concentrations. Bacteria were inoculated at an OD_600_ of 0.01-0.1. Optical density at 600 nm was determined in a microplate reader. For VDC experiments, apical media were serially diluted in PBS and plated on LB (EPEC), MRS (*L. reuteri*) or BHI-YH agar (*R. gnavus*). EPEC plates were incubated overnight at 37°C in air. For *L. reuteri* or *R. gnavus*, plates were placed in an anaerobic cabinet overnight or for 2-3 days, respectively, and CFU were counted. No growth of *L. reuteri* or *R. gnavus* was detected on LB plates cultured under aerobic conditions. For adherence, cell monolayers were washed twice in PBS, lysed in 1% Triton X-100 (v/v) in PBS for 15 min and CFU were quantified as described above. As *R. gnavus* was sensitive to Triton X-100, cell lysates were prepared by vigorous pipetting in PBS.

### Immunofluorescence staining and microscopy

Bacteria and cells were fixed in 3.7% formaldehyde (v/v) in PBS for 10 min at room temperature or in methanol/acetone (1:1) for 4 min on ice for mucin staining. Samples were blocked and permeabilized (if required) in 0.5% bovine serum albumin (w/v) and 0.1% Triton X-100 (v/v) in PBS for 20 min. Specimens were incubated in primary antibodies for 60 min at room temperature. The following antibodies were used in this study: goat anti-*E. coli* (1:400; ab13627, Abcam), mouse anti-*E. coli* LPS (1:200; ab35654, Abcam), rabbit anti-*R. gnavus* NanH (1:200) (Owen et al., 2017), rabbit anti-*L. reuteri* CmbA (1:250) (Etzold et al., 2014), rabbit anti-MUC2 (1:250; sc-15334, Santa Cruz Biotechnology), mouse anti-MUC2 (1:250; sc-7314, Santa Cruz Biotechnology) and rabbit anti-occludin (1:20; 40-4700, Invitrogen). For detection, samples were incubated in donkey anti-rabbit, donkey anti-mouse or donkey anti-goat IgG conjugated with Alexa Fluor 488 or Alexa Fluor 568 (1:400; A10037, A10042, A11057, A21206, A21202, Invitrogen) for 30 min. Actin and nucleic acids were stained with fluorescein isothiocyanate-conjugated phalloidin and 4′,6-diamidino-2-phenylindole (DAPI), respectively. Samples were mounted in Vectashield (Vector Laboratories) and analysed using an Axioimager fluorescent light or LSM800 confocal laser-scanning microscope (Zeiss).

### Western blotting

EPEC effector secretion was determined as described previously (Cameron et al., 2018). Briefly, apical media were centrifuged at 4000 ***g***, 4°C for 10 min. Supernatants were filter sterilized, concentrated 8-fold using Amicon Ultra-4 centrifugal filter units (10 MWCO, Millipore), and 5× reducing sample buffer (RSB) was added. Bacterial pellets were suspended in 1× RSB. After denaturation at 95°C for 5 min, proteins were separated in 12% SDS-polyacrylamide gels (Mini-PROTEAN Tetra Cell, Bio-Rad) for 60 min at 150 V, 100 mA, 10 W. Proteins were transferred to PVDF membranes (Amersham™) by wet blotting at 100 V constant for 60 min. Membranes were blocked in 3% (w/v) bovine serum albumin in Tris-buffered saline with 0.05% (v/v) Tween-20 for 60 min and probed with rabbit anti-EspB (1:1000; Gad Frankel, Imperial College London, UK) (Knutton et al., 1998) or rabbit anti-*E. coli* GroEL (1:50,000; G6532, Sigma-Aldrich) overnight at 4°C. Blots were subsequently incubated with HRP-conjugated goat anti-rabbit IgG (1:200,000; Sigma-Aldrich) for 45 min. Membranes were developed using enhanced chemiluminescence (Pierce™) and imaged with a G:Box Chemi XRG Imager (Syngene). ImageJ Fiji software (https://imagej.net/software/fiji/) was used for densitometric analysis of imaged blots. EspB band intensities were normalized according to signals of the housekeeper protein GroEL.

### IL-8 secretion

Media from apical or basal chambers were sampled and bacteria removed by centrifugation at 4000 ***g*** for 10 min at 4°C. IL-8 concentrations in supernatants were determined with a human IL-8 ELISA kit (PeproTech) according to the manufacturer's instructions.

### Statistical analysis

Data were analysed with GraphPad prism version 9.1.0 (https://www.graphpad.com/scientific-software/prism/). Statistical tests applied are specified in the figure legends, and *P*<0.05 was considered statistically significant.

## Supplementary Material

Supplementary information
