# Supplementary material for: Development of a novel human intestinal model to elucidate the effect of anaerobic commensals on Escherichia coli infection
Source: Dis Model Mech. 2022 Apr 28;15(4):dmm049365. doi: 10.1242/dmm.049365 (PMC9066490; doi:10.1242/dmm.049365)
Supplement: Supplementary information [file dmm-15-049365-s1.pdf]

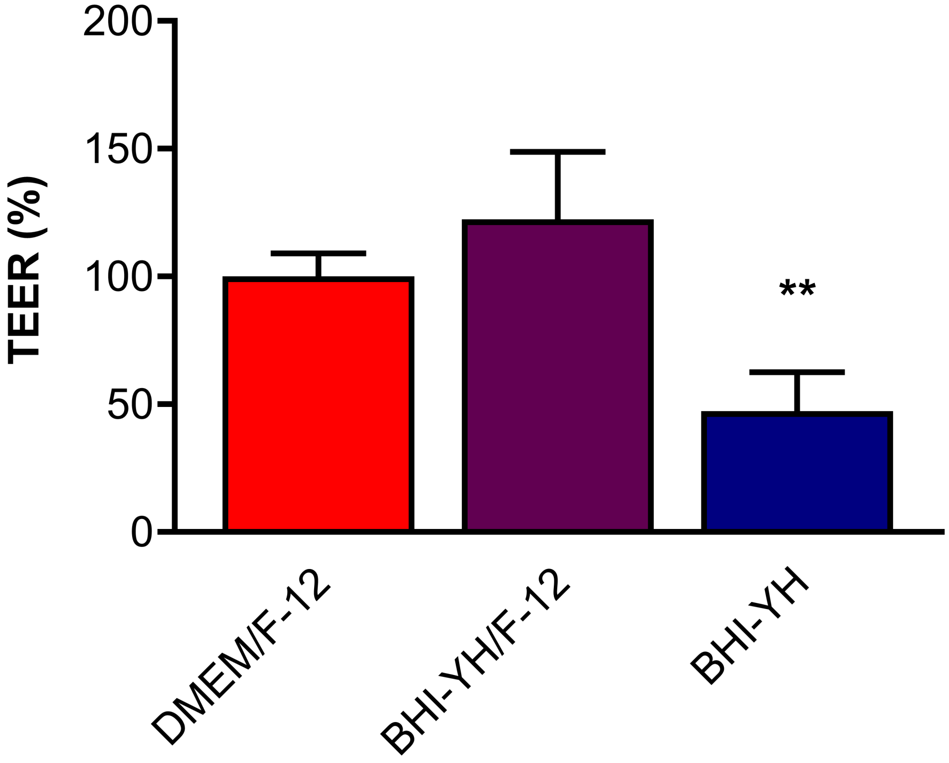

Fig. S1. Influence of BHI-YH medium on T84 epithelial barrier function. Differentiated T84 cells were incubated in the VDC for 22 h with BHI-YH, DMEM/F-12 and a 1:1 mixture of both media (BHI-YH/F-12) on the apical side and DMEM/F-12 on the basal side. TEER was determined before and after incubation and expressed as a percentage change relative to the DMEM/F-12 control. Means ± SD, n = 4. **P<0.01 versus DMEM/F-12 as determined by one-way ANOVA with Dunnett's multiple comparison test.

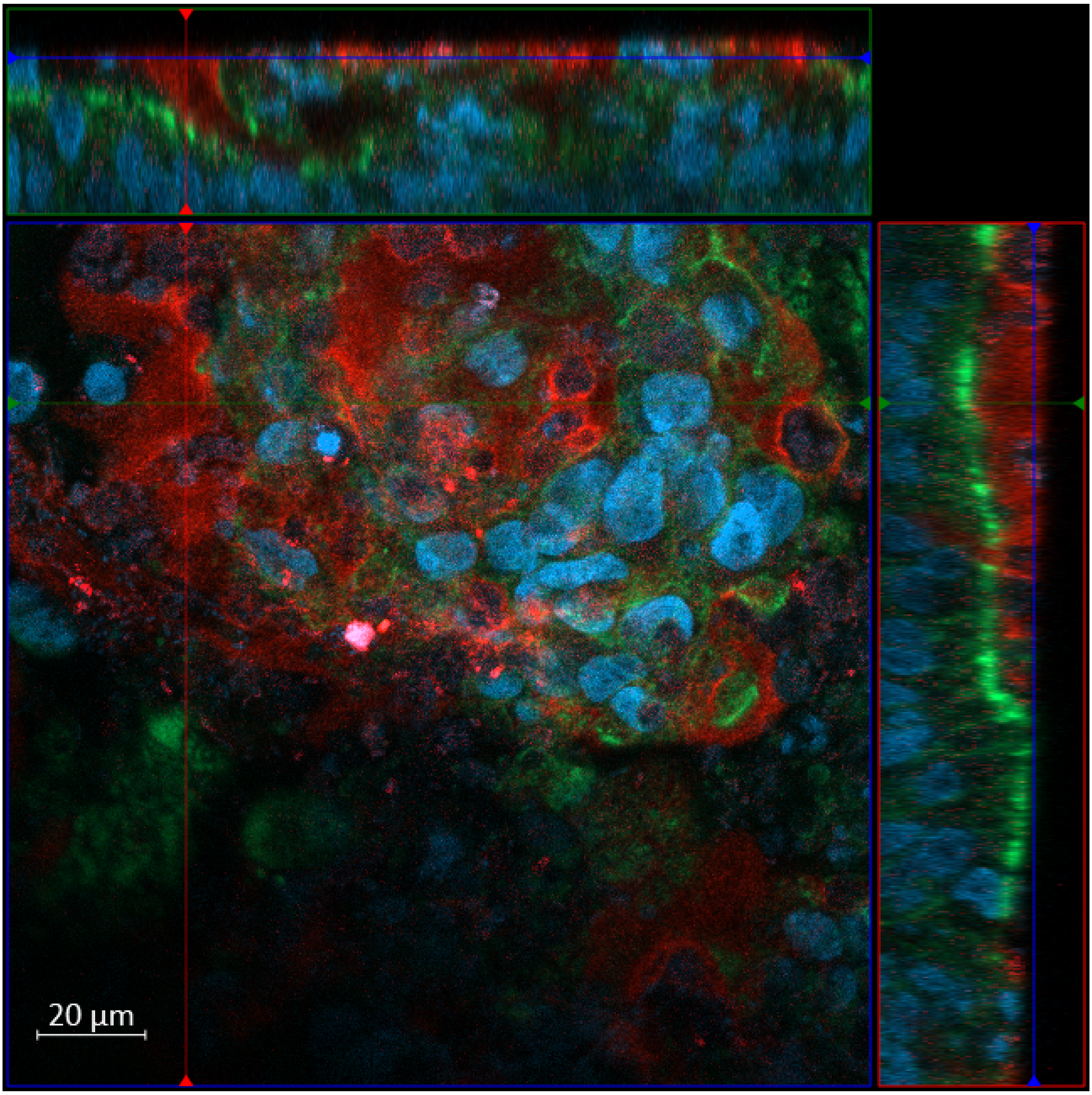

Fig. S2. Orthogonal projection of confocal Z-stack showing mucus secretion by T84/LS174T epithelia. Cells were incubated in the VDC for 4 h and stained for MUC2 (red), F-actin (green) and cell nuclei (blue). Scale bar = 20 µm.

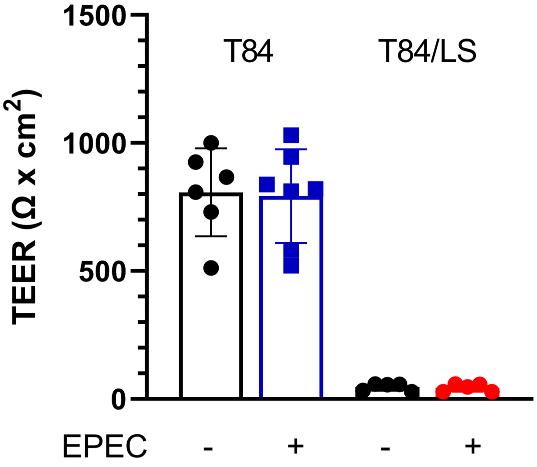

Fig. S3. Transepithelial electrical resistance (TEER) in T84 and T84/LS174T epithelia after EPEC infection. Cell monolayers were incubated with EPEC (+) or left non-infected (-) for 4 h. Medium containing gentamicin (50 µg/ml) was added and incubations continued for 18 h. Shown are individual data points and means ± SD.

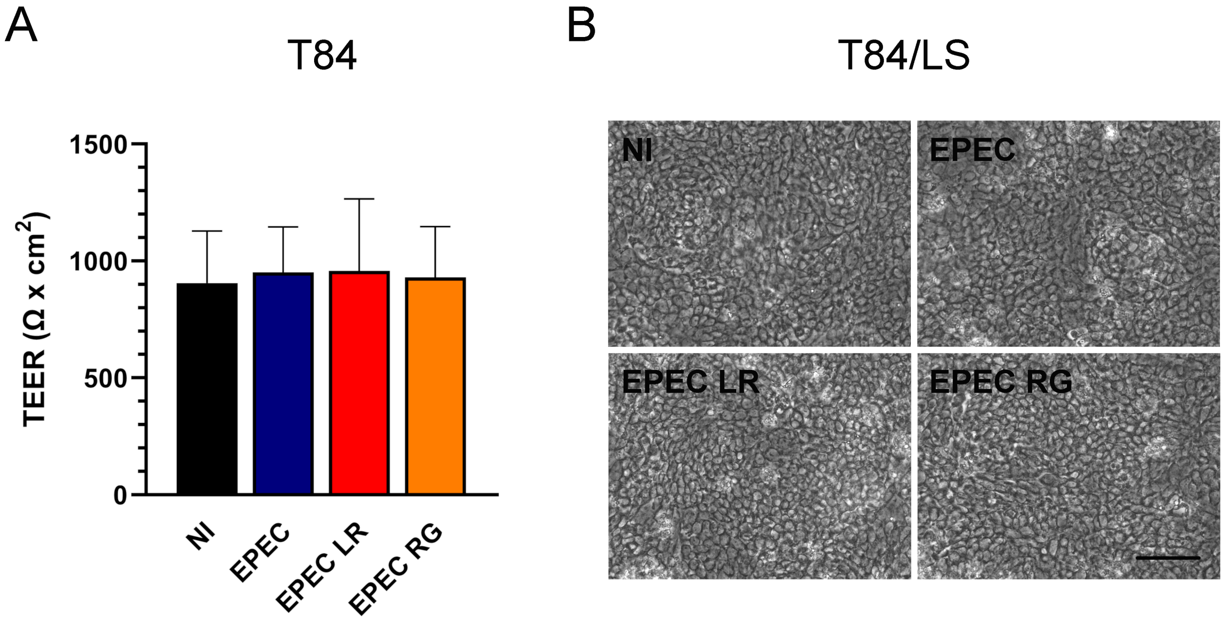

Fig. S4. Maintenance of epithelial integrity during culture with EPEC and commensal bacteria. T84 and T84/LS174T epithelia were incubated with EPEC, EPEC and *L. reuteri* (EPEC LR), EPEC and *R. gnavus* (EPEC RG) or without bacteria (NI) in the VDC for 4 h. (A) Epithelial barrier function of T84 cells was quantified by TEER. Means ± SD, n = 6. (B) Due to low barrier function of T84/LS174T epithelia, monolayer integrity was evaluated by phase contrast microscopy. Scale bar = 50 µm. Images representative of n = 4.

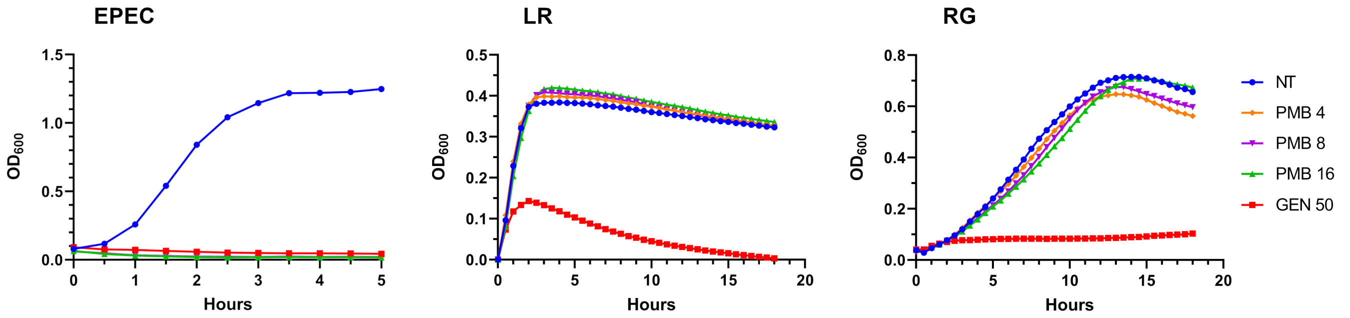

Fig. S5. Antibiotic sensitivity of EPEC, *L. reuteri* (LR) and *R. gnavus* (RG). Bacteria were inoculated in BHI (EPEC), MRS (*L. reuteri*) or BHI-YH (*R. gnavus)* containing polymyxin B (PMB, 4, 8 or 16 µg/ml), gentamicin (GEN, 50 µg/ml) or no antibiotics in 96-well plates. EPEC cultures were maintained under aerobic conditions for 5 h while *L. reuteri* and *R. gnavus* were incubated anaerobically for 18 h. Bacterial growth was monitored every 30 min by optical density (OD600). Shown are means of n = 3 (*L. reuteri* and *R. gnavus*) or n = 6 (EPEC).
